# Polyphosphate-Accumulating Bacteria: Potential Contributors to Mineral Dissolution in the Oral Cavity

**DOI:** 10.1128/AEM.02440-17

**Published:** 2018-03-19

**Authors:** Ashley A. Breiland, Beverly E. Flood, Julia Nikrad, John Bakarich, Matthew Husman, TaekHyun Rhee, Robert S. Jones, Jake V. Bailey

**Affiliations:** a Department of Earth Sciences, University of Minnesota, Minneapolis, Minnesota, USA; b Department of Microbiology, Immunology, and Cancer Biology, University of Minnesota, Minneapolis, Minnesota, USA; c Division of Pediatric Dentistry, School of Dentistry, University of Minnesota, Minneapolis, Minnesota, USA; University of Manchester

**Keywords:** oral biofilms, polyphosphate, dental caries

## Abstract

Bacteria that accumulate polyphosphates have previously been shown to dynamically influence the solubility of phosphatic minerals in marine settings and wastewater. Here, we show that dental plaque, saliva, and carious lesions all contain abundant polyphosphate-accumulating bacteria. Saturation state modeling results, informed by phosphate uptake experiments using the model organism Lactobacillus rhamnosus, which is known to inhabit advanced carious lesions, suggest that polyphosphate accumulation can lead to undersaturated conditions with respect to hydroxyapatite under some oral cavity conditions. The cell densities of polyphosphate-accumulating bacteria we observed in some regions of oral biofilms are comparable to those that produce undersaturated conditions (i.e., those that thermodynamically favor mineral dissolution) in our phosphate uptake experiments with L. rhamnosus. These results suggest that the localized generation of undersaturated conditions by polyphosphate-accumulating bacteria constitutes a new potential mechanism of tooth dissolution that may augment the effects of metabolic acid production.

**IMPORTANCE** Dental caries is a serious public health issue that can have negative impacts on overall quality of life and oral health. The role of oral bacteria in the dissolution of dental enamel and dentin that can result in carious lesions has long been solely ascribed to metabolic acid production. Here, we show that certain oral bacteria may act as a dynamic shunt for phosphate in dental biofilms via the accumulation of a polymer known as polyphosphate—potentially mediating phosphate-dependent conditions such as caries (dental decay).

## INTRODUCTION

The localized ionic saturation state of oral fluids with respect to the thermodynamic solubility product of dental mineral phases influences mineral solubility and the likelihood of enamel dissolution at the tooth/plaque interface or within existing caries lesions (1, 2). Ion exchange between salivary fluids and the tooth surface has widely been accepted as a “chemical” pathway by which Ca^2+^ and PO_4_^3−^, the primary ions in equilibrium with calcium phosphate minerals, are modulated by human-induced, dietary, and nonmicrobial factors in the oral cavity (3). Here, we provide evidence in support of the novel hypothesis that certain oral bacteria may play a considerable role in dynamically modulating the ion concentrations of PO_4_^3−^, and thus the saturation state/solubility of calcium phosphate minerals at the tooth/plaque interface, through intracellular polyphosphate (polyP) accumulation. While all bacteria make short chains of polyP as labile metabolites, polyP-accumulating bacteria (PAB) store substantial intracellular inclusions of polyP in response to specific environmental conditions.

Polyphosphates are linear polymers of orthophosphate residues linked by high-energy phosphoanhydride bonds. polyP accumulation has long been known to be associated with the ability of certain microbes to resist physical and chemical stressors, as well as to provide an alternative source of energy under unfavorable or variable environmental conditions (4–8). The metabolic processes of PAB have been extensively investigated in environmental systems, such as the enhanced removal of phosphorus from wastewater and marine calcium phosphate mineral deposits, which are thought to be mediated by PAB (9–12). It has been demonstrated that PAB are capable of modulating the ionic constituents in equilibrium with apatite group minerals in pore waters and subsequently altering the saturation state of the surrounding fluids, resulting in microenvironments that are thermodynamically favorable for mineral precipitation (12–16). The study of the metabolic processes of PAB in these systems has revealed a new paradigm in our understanding of the modulation of PO_4_^3−^ and Ca^2+^ activities and their relationship to the solubility of calcium phosphate minerals. While previous researchers proposed that the synthesis of polyphosphate by Streptococcus may play a role in caries (17–19), the influence of diverse polyphosphate-accumulating bacteria on mineral saturation state has not, to our knowledge, been applied to the oral environment, where we show that biofilms in plaque and saliva contain abundant polyphosphate-accumulating bacteria that may chemically exchange ions with the calcium phosphate minerals that comprise the inorganic portion of the tooth.

The oral microbiome is a dynamic and diverse community that develops under a wide variety of environmental conditions. Although oral biofilm research has spanned more than one hundred years, little work has been done to investigate the role microbes may play as dynamic mediators of ion concentrations. The current and widely accepted model of the caries process is based on the ability of cariogenic plaque microbiota to establish and thrive in low pH environments in which the metabolic production of mixed acids contributes to enamel demineralization (20–23). Although localized acid production in cariogenic biofilms undoubtedly impacts mineral solubility, the biological influence on chemical saturation of Ca^2+^ and PO_4_^3−^ may present an additional component to the development and rapid progression of carious lesions. To address several different facets of the hypothesis that PAB may affect localized chemical saturation in the oral cavity, we analyzed genomic databases of oral taxa, we quantified PAB in clinical samples of plaque, saliva, and dentinal lesions, we conducted phosphate uptake experiments using a defined *in vitro* single-species model, and we modeled the potential impact of polyP accumulation on the saturation state of saliva.

## RESULTS

### Potential for polyphosphate metabolisms in genomes of oral taxa.

**FIG 1:**
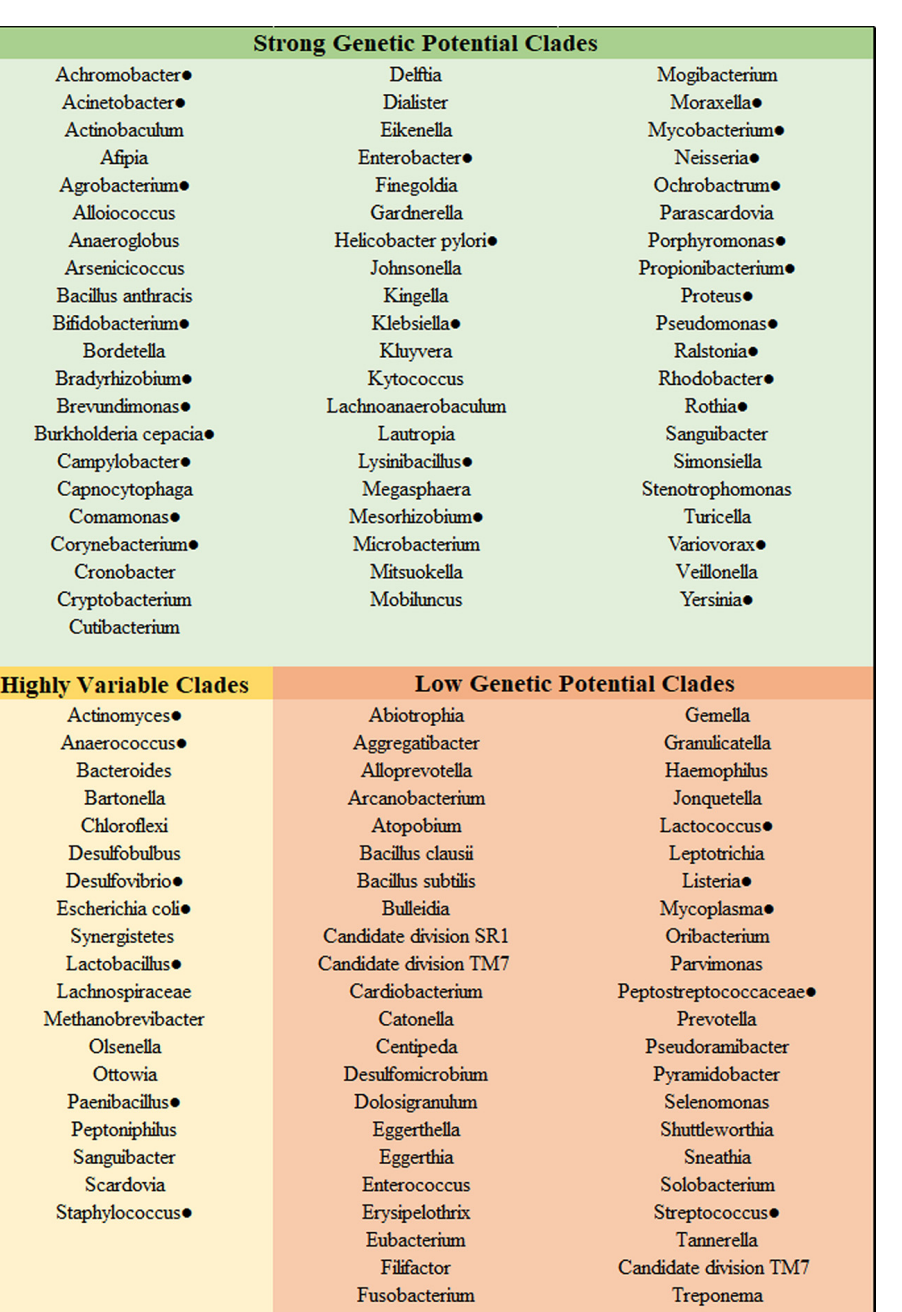
Putative genetic potential to synthesize polyP as defined by the presence of specific genes (see Materials and Methods) in most of the available genomes for that clade (green); some of the available genomes for that clade (yellow) or few/none of the available genomes for that clade (red). See Data Set S1 in the supplemental material for more details. The filled circles indicate there are literature reports of polyP accumulation in those clades.

In bacteria, the main enzymes responsible for synthesizing polyP and subsequently hydrolyzing polyP are polyphosphate kinase (ppk1 and ppk2) and exopolyphosphatase (ppx), respectively (6, 24, 25). We included the associated well-studied genes for hydrolyzing polyP in our search of genomic databases in oral taxa in the Human Oral Microbiome Database (HOMD; see Data Set S1 in the supplemental material) (26, 27). In general, the genetic potential to accumulate polyP was found broadly across the oral microbiome (Fig. 1). Multiple caries-associated clades strongly demonstrated the genetic potential to accumulate polyP, such as Propionibacterium (140/142), Lactobacillus (606/842), Rothia (12/12), Actinomyces (41/76), and Bifidobacterium (291/292). Notably absent were the streptococci, including Streptococcus sobrinus SL-1 (ATCC 33478), which was reported to accumulate polyP (18) (only 29 nonoral isolates of 2,875 genomes). In addition to querying for the primary genes responsible for synthesizing and hydrolyzing polyP, we queried the annotated genomes for the gene encoding polyphosphate glucokinase (*ppgk*) (EC 2.7.1.63) (28). This gene is found in the order Actinomycetales, which includes many clades of caries-associated bacteria (e.g., Actinomyces, Corynebacterium, and Rothia). In this clade, PPGK phosphorylates glucose during its degradation via the Embden-Meyerhof-Parnas pathway. What makes PPGK particularly interesting here is that its phosphoryl donor is often polyphosphate. Our gene survey detected *ppgk* broadly throughout the Actinomycetales as well as some strains of one other known polyphosphate accumulator, Bradyrhizobium spp.

### Oral biofilms contain abundant polyphosphate-accumulating bacteria.

**FIG 2:**
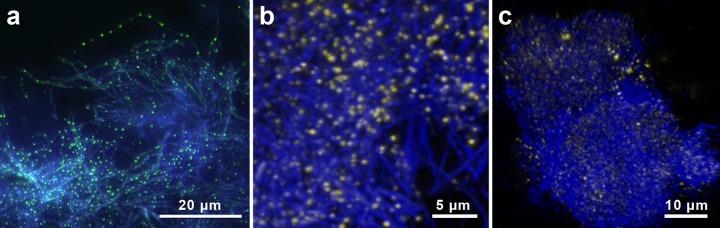
Staining of clinical samples with DAPI (4′,6-diamidino-2-phenylindole). Fluorescence microscopic examination demonstrates that plaque (a and b) and dentinal lesions (c) contain abundant, morphologically diverse, and spatially heterogeneous bacteria that accumulate polyP that can be stained with DAPI (yellow/green inclusions).

**FIG 3:**
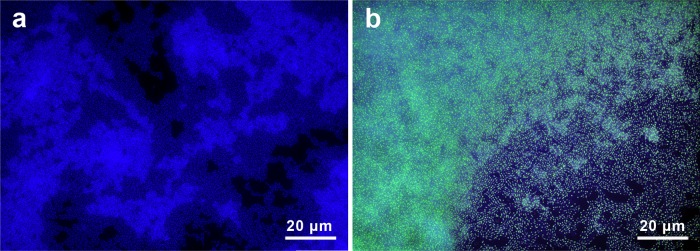
Polyphosphate accumulation in L. rhamnosus in response to nutritional limitations. (a) L. rhamnosus cultured in Mn^2+^-deficient glucose medium for 21 h exhibited no accumulation of polyP. (b) L. rhamnosus cells cultured in “standard” Mn^2+^ glucose medium for 21 h were replete with inclusions of polyP (yellow/green spheres).

Our microscopy observations show that plaque (Fig. 2a), dentinal lesions (Fig. 2c), and our model organism, Lactobacillus rhamnosus (Fig. 3), contain abundant intracellular polyP inclusions that can be visualized with the DNA stain DAPI (4′,6-diamidino-2-phenylindole). The binding of polyP to DAPI shifts its peak emission wavelength from 475 nm (blue for DNA) to 525 to 550 nm, at an excitation wavelength of 360 nm, where the DAPI-polyP complex appears yellow and inclusions can be observed as discrete yellow/green spheres within the cell (Fig. 2) (29). We found that the dental plaque samples from all 30 patients sampled (60 samples in total) contained polyP inclusion bodies in morphologically diverse and spatially heterogeneous oral biofilms. The staining of dentinal lesions, from five extracted teeth, also revealed abundant polyP inclusion bodies. As expected, the bacterial morphotypes in the dental plaque samples include long filamentous organisms as well as small cocci and bacillus-shaped cells. The morphological diversity of the dentin samples appeared to be less than that of the dental plaque, primarily consisting of small cocci and bacillus-shaped organisms, while filamentous bacteria were absent.

We used spectral-scanning confocal microscopy to resolve polyP inclusions in complex three-dimensional oral biofilms by linearly unmixing the spectral signal for polyP-DAPI from that of DNA-DAPI (Fig. 2b and c; see also Fig. S1). polyP inclusions were not homogenously dispersed throughout the biofilms and appeared to be denser within certain regions of the sample. In some regions, the cell densities observed in the oral biofilms exceeded those of L. rhamnosus in our phosphate uptake experiments, reaching up to 1.06 × 10^12^ inclusions/cm^3^, which led us to hypothesize that these dense assemblages can conceivably modulate localized saturation gradients to create undersaturated conditions within discrete areas of the biofilm.

### Polyphosphate accumulation in Lactobacillus rhamnosus.

We selected the caries-associated bacterium Lactobacillus rhamnosus as a model organism to manipulate and observe polyP metabolisms in response to nutritional limitations and dynamic environmental conditions because of its ability to accumulate polyP and because it is associated with the progression of dental caries (25). When grown on a semidefined medium, L. rhamnosus accumulated polyP within a 24-hour period, but only when manganese (0.05 g/liter) was included. By comparing a Mn^2+^-amended culture to an unamended culture, we were able to quantify the influence of polyP accumulation on the extracellular phosphate concentration.

### Colorimetric inorganic phosphate quantification.

By developing a medium that enables us to grow L. rhamnosus under conditions that allow for polyP accumulation versus conditions that do not, we were able to spectrophotometrically quantify the relative concentration of phosphorus from our medium that is being incorporated as intracellular polyP inclusions. Using a colorimetric assay (30), we compared the supernatants of our L. rhamnosus cultures to a known set of phosphate standards to quantify the depletion of total inorganic phosphate during the growth cycle.

**FIG 4:**
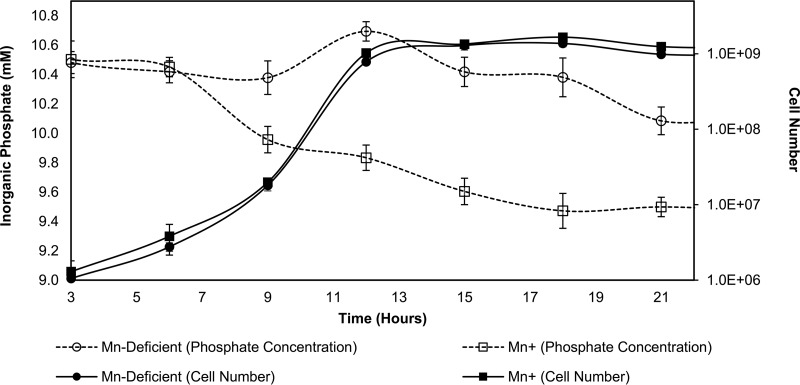
Inorganic phosphate quantification using the oral isolate L. rhamnosus. Growth of L. rhamnosus under conditions that allow for polyP accumulation (“standard” Mn^2+^) resulted in a decrease of P_i_ in the medium of approximately 1.3 mM at 18 h. L. rhamnosus grown without the addition of Mn^2+^, which prevents polyP accumulation (Mn^2+^ deficient), resulted in a decrease of P_i_ in the medium of approximately 0.4 mM. Initial medium P_i_ concentration was approximately 10.8 mM. Error bars indicate ± one standard deviation from the mean.

Since bacteria use phosphate for a variety of different purposes other than polyP accumulation, it is important to take into consideration the quantity of phosphorus necessary for cell growth. Figure 4 illustrates inorganic phosphate changes, over the course of 24 h, between our Mn^2+^-deficient glucose medium (negative polyP accumulation) and “standard” Mn^2+^ glucose medium (positive polyP accumulation). L. rhamnosus grown under conditions that allow for polyP accumulation (“standard” Mn^2+^) shows a decrease in medium PO_4_^3−^ concentrations of approximately 1.3 mM after 18 h of incubation. L. rhamnosus grown in Mn^2+^-deficient glucose medium, i.e., Mn^2+^-limited conditions that prevent polyP accumulation, shows a decrease of approximately 0.38 mM PO_4_^3−^ after 18 h of incubation. We make the assumption that most, if not all, of the difference in the total PO_4_^3−^ concentrations results from P_i_ uptake and storage in the cells as the intracellular polyP granules that we observed in the standard Mn^2+^ cultures. Since the cell densities between the two media were similar (Fig. 4), with the Mn^2+^-deficient culture being slightly less dense, the concentration of phosphate was adjusted to reflect equal cell densities among the two media. Using this assumption, we calculate a maximum net change of phosphate via polyP accumulation by L. rhamnosus to be approximately 0.91 (±0.12) mM after 18 h of incubation. If polyP-accumulating bacteria in plaque or dentinal lesions are accumulating similar amounts of polyP as our model organism, L. rhamnosus, is capable of accumulating, then it is probable that oral biofilms influence the saturation chemistry of the saliva/mineral interface through the metabolism of polyP. To assess the potential impact polyP accumulation and/or release may have on mineral solubility, we used a Web-based geochemical modeling program, WEB-PHREEQ (https://www.ndsu.edu/webphreeq/), based on the program PHREEQC (31), to calculate changes that would result in supersaturated or undersaturated conditions with respect to the apatite group mineral hydroxyapatite.

### Saturation state response to polyphosphate accumulation.

One of the primary factors controlling the solubility of hydroxyapatite in dental enamel or dentin is the saturation state of saliva with respect to its primary ionic constituents, calcium and phosphate (32). The saturation state refers to the thermodynamic dependence of the mineral's solubility on the product of the activities (i.e., functional concentrations) of the constituent ions in equilibrium with the solid phase (e.g., the inorganic portion of the tooth enamel). For undersaturated conditions (Ω < 1), the dissolution of the mineral is thermodynamically favored, while under supersaturated conditions (Ω > 1), mineral precipitation is favored. Dissolving table salt (NaCl) in a dilute solution is an example of mineral dissolution in an undersaturated solution. Despite the fact that saliva is generally supersaturated with respect to hydroxyapatite (32, 33), some individuals experience extensive mineral dissolution while others accumulate dental calculus (mineralized plaque).

**FIG 5:**
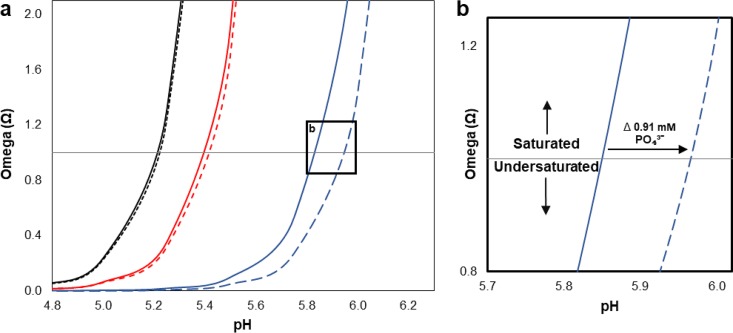
Modeled shift in relative saturation of salivary fluids in response to bacterial polyP accumulation. (a) Solid lines represent the saturation state modeled on values derived from literature for salivary concentrations of PO_4_^3−^ and Ca^2+^ (34). High PO_4_^3−^ and Ca^2+^ values of 12.6 and 4.2 mM, respectively (black curve), medium PO_4_^3−^ and Ca^2+^ values of 7.3 mM and 2.65 mM, respectively (red curve), and low PO_4_^3−^ and Ca^2+^ values of 2.0 mM and 1.1, mM, respectively (blue curve). (b) Corresponding dashed lines represent a shift in relative saturation in response to polyP accumulation resulting in an ∼0.91 mM PO_4_^3−^ concentration change (arrow), as determined by our single-species phosphate uptake model of L. rhamnosus. A PO_4_^3−^ drawdown of 0.91 mM changes the saturation state to one in which dissolution becomes thermodynamically favorable at a higher pH than without the drawdown in a scenario where PO_4_^3−^ and Ca^2+^ concentrations are low in the surrounding fluids. Relative saturation equilibrium (Ω = 1) is represented by the solid horizontal line. Omega values of >1 represent saturated conditions, while omega values of <1 represent undersaturated conditions.

We modeled the mineral saturation state of saliva using three different salivary concentrations of calcium and phosphate (34) and compared these saturation states with those in which we subtracted the 0.91 mM phosphate derived from our L. rhamonosus phosphate uptake experiments (Fig. 5). From these results, we conclude that when salivary calcium and phosphate concentrations are high (4.2 and 12.6 mM, respectively), the impact on saturation state resulting from a phosphate drawdown of 0.91 mM is minimal. However, when calcium and phosphate concentrations in the saliva are relatively low (1.1 and 2.0 mM, respectively), a drawdown of 0.91 mM phosphate can have a sizable effect on mineral solubility. If this magnitude of change were to occur in saliva at pH 5.85 (assuming a stable calcium concentration of 1.0 mM and an initial phosphate concentration of 2.0 mM), then the system would go from saturated with respect to hydroxyapatite (Ω = ∼1) to undersaturated (Ω = ∼0.50), a condition that thermodynamically favors mineral dissolution.

## DISCUSSION

Dental caries is a dynamic and multifactorial disease whose etiology is thought to be based largely on the ability of the bacterial community to produce acid and survive under decreasing pH conditions (35). Tooth enamel, primarily composed of hydroxyapatite [Ca_5_(PO_4_)_3_(OH)], is highly susceptible to demineralization from prolonged exposure to organic acids, by-products of bacterial carbohydrate fermentation (22). Streptococcus mutans was established early on as a key player in the caries process and remains a focus of investigation due to its acidogenic and aciduric properties (35, 36). However, recent studies have shown that although S. mutans is associated with both enamel and dentinal carious lesions, it is part of a larger consortium of cariogenic bacteria that thrive under low pH conditions as a result of frequent carbohydrate exposure (20, 22, 23). Recent developments in community characterization using 16S rRNA gene-based amplicon sequencing and metagenomics have made it possible to identify and study the microbiota associated with cariogenic plaque. Along with S. mutans, cariogenic plaque is composed of a community of diverse microbial species, including Rothia, Actinomyces, Bifidobacterium, Lactobacillus spp., and other nonmutans streptococci (20, 35, 37–39). Among the microbiota identified as key players in the development of carious lesions, our genome analysis results show that several clades possess the genetic potential to accumulate polyP. In most of the caries-associated clades, the capacity to accumulate polyP has also been demonstrated experimentally (25, 40, 41). Whether strains of Streptococcus, in particular S. mutans, have the capacity to accumulate polyP by a yet-to-be-identified genetic pathway remains to be explored. Regardless, the capacity to accumulate and release polyP appears in many cases to be highly variable within many clades, even at the strain level, and may contribute to the phenotypic variability within caries microbiomes, resulting in the modulation of disease progression. For example, the acid-producing fermentation of sugar, the canonical primary driver of caries, when directly coupled to polyphosphate metabolism in strains with polyphosphate glucokinase, could result in the concomitant production of acid and the reduction of the saturation state of oral fluids via phosphate uptake.

Our preliminary results from DAPI staining 60 plaque, five carious dentin, and 60 saliva samples demonstrate that PAB are ubiquitous in the oral cavity (123/123). Even though many of the organisms we observed to contain accumulations of polyP in the oral cavity may not be associated with the development and progression of dental caries (i.e., certain plaque and salivary microbiota), the mere presence of heterogeneous dense assemblages of PAB in the oral community conceivably introduces a new paradigm in the realm of dental disease and oral microbial ecology. PAB present in our clinical samples of dentinal lesions provide suggestive evidence that polyP accumulation may play a role in P_i_ modulation between bacteria and dental enamel. Since we were unable to identify which species of PAB were present within these dentinal lesions, we utilized *in vitro* culturing of L. rhamnosus, an organism known to be associated with caries progression, to determine if polyP accumulation may have an effect on the saturation state of the surrounding fluids in an environment where PAB are well established.

Our results demonstrate that L. rhamnosus accumulates concentrations of polyP that could result in changes in saturation state to the surrounding oral environment in situations where PO_4_^3−^ concentrations are naturally low or depleted at the tooth/biofilm interface. Unrecognized factors such as dietary nutrient sources, continued organic acid exposure, and oxygenation conditions may initiate bacterial polyP uptake and reduce (or increase) localized concentrations of Ca^2+^ and PO_4_^3−^. The uptake and subsequent release of inclusions of polyP may significantly alter the saturation chemistry of the fluids surrounding the tooth surface by shifting the chemical equilibrium and result in dissolution of apatite group minerals in the form of dental caries.

**FIG 6:**
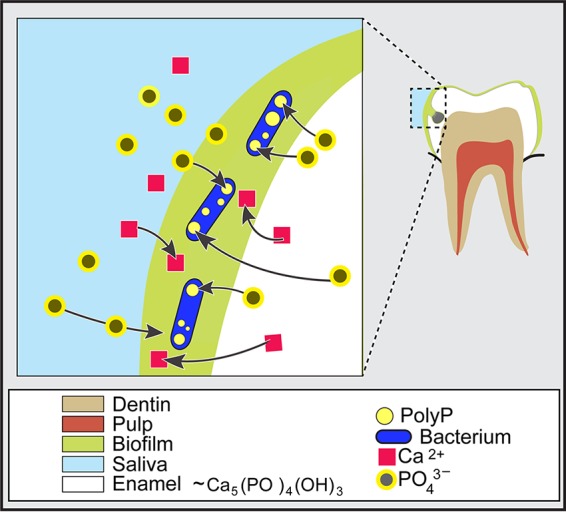
Schematic of polyphosphate-accumulating bacteria taking up inorganic phosphate to synthesize polyP potentially resulting in undersaturated conditions that lead to mineral dissolution and caries progression. Polyphosphate-accumulating bacteria may also acquire PO_4_^3−^ from acid-induced dissolution of the enamel.

In a scenario where nutrient availability is limited, the pH is relatively acidic, and oxygen levels are depleted, PAB such as L. rhamnosus may exist as opportunists that establish themselves in an exclusive ecological niche in which their polyP metabolisms may provide a competitive edge among other oral microbiota. Carious lesions that had initially demineralized from exposure to mixed organic acids may become even more susceptible to mineral loss as aciduric/acidogenic PAB, such as L. rhamnosus, establish themselves in the vicinity of dissolution. As tooth enamel demineralizes, PAB may increase dissolution by further disrupting the chemical balance of Ca^2+^ and PO_4_^3−^ by accumulating P_i_ from the dissolved enamel, thus creating a demineralization environment that results both from acid production and phosphate depletion (Fig. 6).

The sequestration of PO_4_^3−^ by PAB has the potential to alter the chemical conditions of the oral environment that promote mineral dissolution under certain conditions in the mouth, leading to dental decay. Alternatively, the concentrated release of PO_4_^3−^ from PAB could lead to the precipitation of dental calculus (mineralized dental plaque) under a different set of oral microenvironmental conditions. These ions can also be incorporated into various other phases of apatite such as fluorapatite [Ca_5_(PO_4_)_3_F] and carbonate hydroxyapatite [Ca_5_(PO_4_,CO_3_)_3_(OH)]. These substitutions are common in the oral cavity and vary from individual to individual, as well as from tooth to tooth. Mineral solubility may increase or decrease depending on the substitutions in the lattice structure (42). To assess the controls on PAB metabolisms and their potential roles in altering the saturation chemistry of the saliva/mineral interface, a comprehensive understanding of the ecology and physiology of PAB in the oral environment is needed. Clinical assessments and *in situ* taxonomic identification of PAB in oral biofilms will aid us in understanding their ecophysiologies, as well as aid our ability to treat oral diseases such as dental caries that remain incompletely understood.

## MATERIALS AND METHODS

### Genomic identification of candidate isolates.

An initial list of 1,362 microbial genomes of oral taxa in the HOMD was used for detecting genes related to polyP accumulation in oral microbes. With the exception of that for one strain of Aggregatibacter, the genome for each HOMD taxon was available and annotated by the DOE's integrated microbial genome pipeline (IMG/M) (43). The genomes were queried (find function search) for genes classified as coding for the enzymes PPK1 (EC 2.7.4.1), PPX (EC 3.6.1.11), PPGK (EC 2.7.1.63), and COG PPK2 (COG2326). For a better context of the likelihood that a clade may or may not be a polyP accumulator, genomes of all other strains within the same clade were included in our search for the presence or absence of the queried genes. The results are presented in Data Set S1 in the supplemental material. A few HOMD strains from the list were determined to be duplicate strains and are reported as such. When available, peer-reviewed studies were utilized in the interpretation of genetic potential. Complete references cited in Data Set S1 are located in the supplemental material.

### Lactobacillus rhamnosus phosphate uptake experiments.

Lactobacillus rhamnosus ATCC 7469 (DSM 20021), obtained from the USDA Agricultural Research Service Culture Collection, was used in our single-species model to assess polyP metabolic potential in a caries-associated organism. Specifically, the growth of L. rhamnosus under conditions that allow it to accumulate polyP versus under conditions that do not enables us to quantify PO_4_^3−^ uptake specific to polyP that we then used to model saturation state changes under conditions in which fluctuations in intracellular polyP accumulation may affect oral saturation state chemistry. Two media modified after a Lactobacillus MRS growth medium (44) were developed containing the following (g/liter): 20 d-(+)-glucose monohydrate, 99%; 10 peptone type 1; 5 yeast extract; 5 sodium acetate; 0.1 MgSO_4_; 2 K_2_HPO_4_; 0.05 MnSO_4_·H_2_O; 1 ml Tween 80. The second medium, designated Mn^2+^-deficient glucose medium, contained the same chemical proportions of the medium described above (“standard” Mn^2+^ glucose medium), with the exception of MnSO_4_·H_2_O, which was excluded from the medium. Triplicate cultures of L. rhamnosus (starting inoculum adjusted to an optical density of 0.2) were cultivated in an aerobic environment for 24 h at 37°C with orbital shaking at 90 rpm. One milliliter of culture was collected from each replicate every 3 h, optical density readings were collected, and samples were centrifuged at 10,000 × *g* for 10 min at 4°C. The supernatants were transferred to separate centrifuge tubes for further chemical analysis, and the cell pellets were resuspended in one milliliter of 50% ethanol for fluorescence microscopic examination after staining for polyP (as described below). After testing for the suitability to preserve polyP granules, ethanol fixation was used to keep cellular structures intact and metabolisms inert.

### Plaque and dentin sampling/collection.

Plaque samples were collected from male or female children between the ages of 4 and 18 years who satisfied one of the following inclusion criteria: (i) had an oral health with an absence of dental caries or hardened dental plaque, (ii) had dental caries or a recent history of dental caries, or (iii) had hardened dental plaque with an absence of dental caries. The process of collecting the dental plaques was in accordance with Institutional Review Board (IRB) procedures at the University of Minnesota (no. 1507M75441). Plaque samples consisted of two separate samples collected from the anterior and posterior dentition using a sterile dental scaler. Two samples were taken from 30 patients for a total of 60 samples.

Prior to the appointment, subjects were expected to have fasted 1 h prior to sampling as well as have refrained from brushing their teeth the morning of sampling. Each sample was placed in a separate test tube containing 1 ml of 50% ethanol. The test tubes were immediately placed on ice for transport and stored at −20°C for future microscopic analysis. Five dentin samples were collected from extracted teeth that were to be discarded as pathological waste. Once the teeth were extracted, gross debris consisting of heme, remnants of periodontal ligament (PDL)/gingival attachments, and any granulomatous tissue was removed chairside with 2 by 2 cotton gauze. Dentin was extracted from each of the carious teeth with sterile hand instrumentation under a class II/type A2 biosafety cabinet. The dentin samples were immediately stained for microscopic visualization and the extracted teeth were placed into individual tubes containing 50% ethanol.

### Polyphosphate identification via fluorescence microscopy.

DAPI binds to both polyP and DNA, and the corresponding complexes, polyP-DAPI complex and the DNA-DAPI complex, have distinct emission spectra (461 nm and 525 nm, respectively) when excited by 360-nm light. To resolve the polyP-DAPI complex, custom band-pass filters (Chroma) were employed (DNA-DAPI excitation/emission, 345/455 nm; polyP-DAPI excitation/emission, 415/550 nm). This emission wavelength shift results in the emission of a distinct yellow color that can be used to differentiate the polyP-DAPI complex from the DNA-DAPI complex (45). Nine samples of L. rhamnosus, collected every 3 h over the course of a 24-h period, as well as 60 plaque and five dentin samples, were collected and fixed for staining. Each ethanol-fixed sample was placed in a designated well on a Teflon-printed microscope slide and allowed to air dry until the cells were adhered to the slide. Eight microliters of 5-μg/ml DAPI was pipetted onto each sample-containing well and left to incubate in the dark for 30 min in a hybridization chamber. Imaging was performed on an Olympus BX61 fluorescence microscope equipped with an XM10 charge-coupled-device (CCD) camera and cellSens Dimensions imaging software (v. 1.13). Photoshop CS5 (v. 12.0.4) was used to adjust the brightness and contrast uniformly across the entirety of images in Fig. 3a and b.

### Confocal spectral imaging.

Confocal spectral imaging was employed using an Olympus Fluoview FV1000 IX2 inverted confocal microscope. Olympus Fluoview (v. 04.01.01.05) was employed for image acquisition. Linear spectral unmixing of the polyP-DAPI from the DNA-DAPI signal was performed by designating regions of interest based on emission properties obtained from a spectral wavelength lambda scan of the sample. The sample was scanned from 450 nm to 580 nm in increments of 10 nm. Ethanol-fixed oral biofilm samples were stained with DAPI and analyzed under a 60× oil objective with a numerical aperture of 1.42. polyP and DNA signals were separated based on a standard polyP emission wavelength (550 nm) and DNA emission wavelength (461 nm).

Images were analyzed with AutoQuant X (v. X3.0.4) and Imaris x64 (v. 7.5.2). Blind three-dimensional deconvolution with default settings (adaptive point spread function [PSF], 10 iterations, and medium noise) was employed in AutoQuant X. After image deconvolution, the files were exported to Imaris x64 for three-dimensional analysis and polyP quantification. polyP inclusions were quantified by creating a three-dimensional surface characterized by the emission spectra of polyP-DAPI. Designated regions of interest (ROI) with substantial densities of polyP inclusions were selected to quantify the amount of polyP-containing cells within a dense region. Regions of interest ranged in size from 5 μm^3^ to 10 μm^3^ (see Fig. S2 for an example ROI surface estimation).

### Inorganic phosphate quantification.

An inorganic phosphate (Pi) quantification method (30) was adapted (100 μl ascorbic acid-mixed reagent to 1.0 ml of sample) to assess the influence of polyP accumulation on the P_i_ concentration in the extracellular medium in our model organism, L. rhamnosus. To account for the amount of phosphate that would have been taken up from the medium for purposes other than polyP accumulation, our semidefined medium was modified to inhibit cellular polyP accumulation while maintaining cell densities similar to those of the polyP-accumulating culture. Cell counts from three experimental replicates were used to normalize the slightly different cell densities between the two culture types when calculating phosphate change/cell.

### Geochemical analysis and saturation calculations.

To assess the potential impact cellular polyP accumulation/release has on enamel mineral solubility (i.e., hydroxyapatite), we employed WEB-PHREEQ: aqueous geochemical modeling (version 2) to model chemical saturation state fluctuations in response to fluctuating phosphate concentrations. A range of salivary phosphate and calcium concentrations (mM) reported in the literature (34) were used as a series of arbitrary, but patient-derived, starting values in determining the chemical saturation of hydroxyapatite in response to fluctuating pH and phosphate concentrations. Net polyP accumulation (0.91 mM), as previously determined from our L. rhamnosus phosphate uptake experiments, was subtracted from literature-reported phosphate values to assess saturation index fluctuations during our maximum observed cellular polyP accumulation and release.

## Supplementary Material

Supplemental material
