# Supplementary material for: Polyphosphate-Accumulating Bacteria: Potential Contributors to Mineral Dissolution in the Oral Cavity
Source: Appl Environ Microbiol. 2018 Mar 19;84(7):e02440-17. doi: 10.1128/AEM.02440-17 (PMC5861820; doi:10.1128/AEM.02440-17)
Supplement: Supplemental material [file supp_84_7_e02440-17__index.html]

Supplemental material 

# Polyphosphate-Accumulating Bacteria: Potential Contributors to Mineral Dissolution in the Oral Cavity

## Supplemental material

- Supplemental file 1 -

  Spectral properties from confocal microscopy spectral unmixing of oral biofilms (Fig. S1); three-dimensional rendering of z-stack of spectral images of oral biofilm extracted from dentin (Fig. S2); references.

  PDF, 549K
- Supplemental file 2 -

  Numbers of annotated genes related to polyphosphate accumulation detected by the IMG pipeline of strains in the HOMD database (Data Set S1).

  XLSX, 1.1M
