# Supplementary material for: Polyphosphate-Accumulating Bacteria: Potential Contributors to Mineral Dissolution in the Oral Cavity
Source: Appl Environ Microbiol. 2018 Mar 19;84(7):e02440-17. doi: 10.1128/AEM.02440-17 (PMC5861820; doi:10.1128/AEM.02440-17)
Supplement: Supplemental material [file AEM.02440-17_zam007188396s1.pdf]

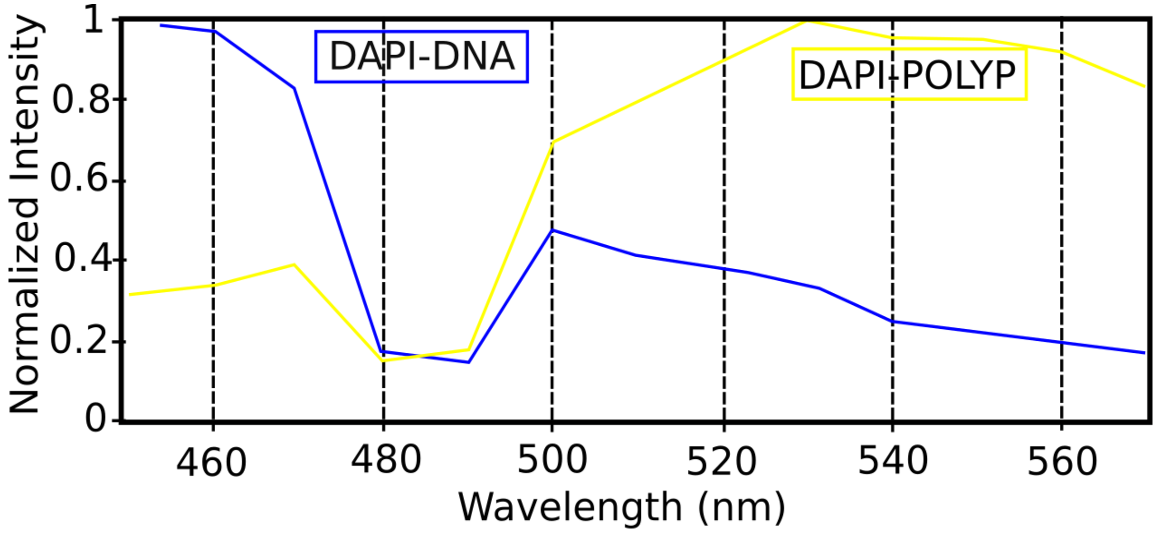

Fig. S1 | Spectral properties from confocal microscopy spectral unmixing of oral biofilms. DAPI-DNA complex (blue line) peak emission at approximately 460 nm. DAPI-PolyP complex (yellow line) peak emission at approximately 530 nm.

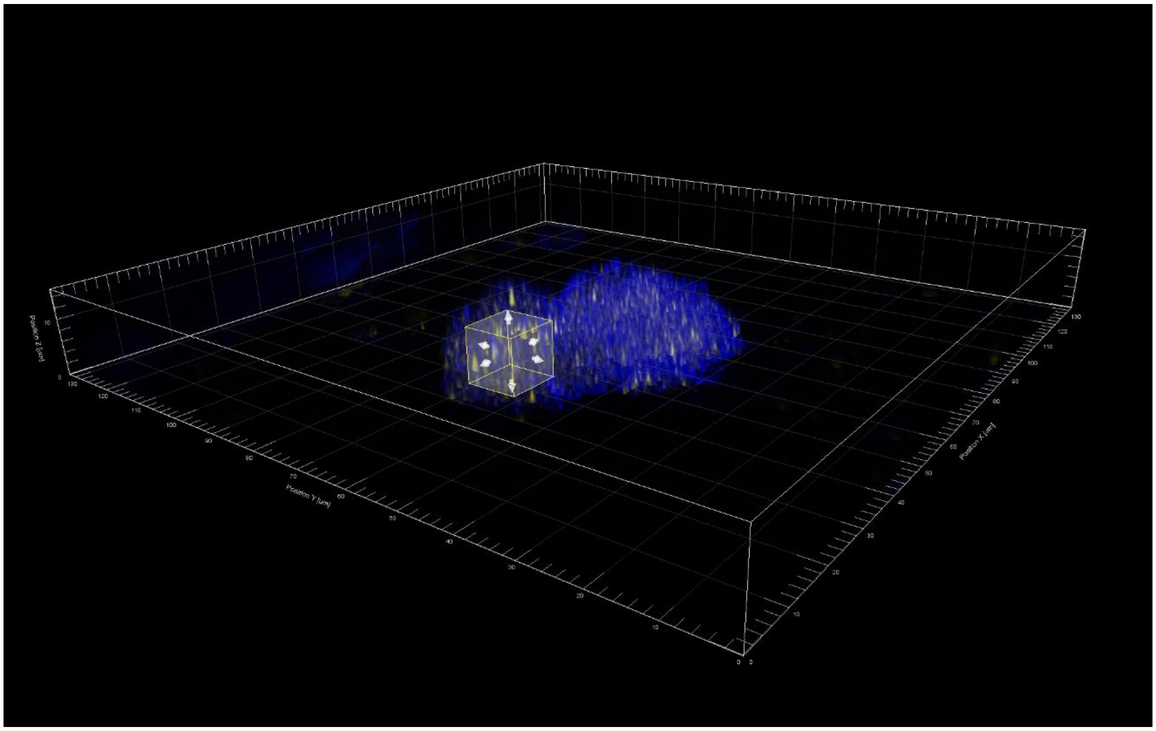

Fig. S2 | Three-dimensional rendering of z-stack of spectral images of oral biofilm extracted from dentin. Polyphosphate surfaces were counted within designated 10μm^3 volumes (i.e. transparent cube near center of image).

SI Text References

1. Aiking H, Stijnman A, Van Garderen C, Van Heerikhuizen H, Van't Riet J. 1984. Inorganic phosphate accumulation and cadmium detoxification in *Klebsiella aerogenes* NCTC 418 growing in continuous culture. Appl Environ Microb 47:374-377.
2. Aprea G, Mullan W, Mullan A, Murru N, Tozzi M, Cortesi M. 2005. Isolation of polyphosphate-accumulating lactic acid bacteria from natural whey starters. Milchwissenschaft 60:256-258.
3. Archibald FS, Fridovich I. 1982. Investigations of the state of the manganese in *Lactobacillus plantarum*. Arch Biochem Biophys 215:589-596.
4. Auling G, Pilz F, Busse H, Karrasch S, Streichan M, Schön G. 1991. Analysis of the polyphosphate-accumulating microflora in phosphorus-eliminating, anaerobic-aerobic activated sludge systems by using diaminopropane as a biomarker for rapid estimation of *Acinetobacter spp*. Appl Environ Microb 57:3585-3592.
5. Ault-Riché D, Fraley CD, Tzeng C-M, Kornberg A. 1998. Novel assay reveals multiple pathways regulating stress-induced accumulations of inorganic polyphosphate in *Escherichia coli*. J Bacteriol 180:1841-1847.
6. Bode G, Mauch F, Ditschuneit H, Malfertheiner P. 1993. Identification of structures containing polyphosphate in *Helicobacter pylori*. Microbiology 139:3029-3033.
7. Buzoleva L, Krivosheeva A, Isachenko A, Somova L, Somov G. 2006. Effect of temperature on synthesis of polyphosphates in *Yersinia pseudotuberculosis* and *Listeria monocytogenes* under starvation conditions. Biochemistry (Moscow) 71:437-440.

8. Candon HL, Allan BJ, Fraley CD, Gaynor EC. 2007. Polyphosphate kinase 1 is a pathogenesis determinant in *Campylobacter jejuni*. J Bacteriol 189:8099-8108.

9. Chen W, Palmer RJ, Kuramitsu HK. 2002. Role of polyphosphate kinase in biofilm formation by *Porphyromonas gingivalis*. Infect Immun 70:4708-4715.

10. Clark JE, Beegen H, Wood HG. 1986. Isolation of intact chains of polyphosphate from" *Propionibacterium shermanii*" grown on glucose or lactate. J Bacteriol 168:1212-1219.

11. Cooney CL, Wang DIC. 1976. Transient response of *Enterobacter aerogenes* under a dual nutrient limitation in a chemostat. Biotechnol Bioeng 18:189-198.

12. Costalonga M, Herzberg MC. 2014. The oral microbiome and the immunobiology of periodontal disease and caries. Immunol Lett 162:22-38.

13. Dul'tseva N, Chernitsina S, Zemskaya T. 2012. Isolation of bacteria of the genus *Variovorax* from the *Thioploca* mats of Lake Baikal. Microbiology 81:67-78.

14. Geerligs G, Aldrich H, Harder W, Diekert G. 1987. Isolation and characterization of a carbon monoxide utilizing strain of the acetogen *Peptostreptococcus productus*. Arch Microbiol 148:305-313.

15. Gonzalez H, Jensen T. 1998. Nickel sequestering by polyphosphate bodies in *Staphylococcus aureus*. Microbios 93:179-185.

16. Hiraishi A, Yanase A, Kitamura H. 1991. Polyphosphate accumulation by *Rhodobacter sphaeroides* grown under different environmental conditions with special emphasis on the effect of external phosphate concentrations. Bull Jpn Soc Micro Ecol 6:25-32.

17. Jones H, Chambers LA. 1975. Localized intracellular polyphosphate formation by *Desulfovibrio gigas*. Microbiology 89:67-72.

18. Klauth P, Pallerla SR, Vidaurre D, Ralfs C, Wendisch VF, Schoberth SM. 2006. Determination of soluble and granular inorganic polyphosphate in *Corynebacterium glutamicum*. Appl Microbiol Biotechnol 72:1099-1106.

19. Kostanjšek R, Miloš V, Srot V, van Aken PA, Jasna Š. 2017. Polyphosphate-accumulating bacterial community colonizing the calcium bodies of terrestrial isopod crustaceans *Titanethes albus* and *Hyloniscus riparius*. FEMS Microbiol Ecol 93.

20. Lai C-H, Listgarten M. 1980. Comparative ultrastructure of certain *Actinomyces* species, *Arachnia*, *Bacterionema* and *Rothia*. J Periodontol 51:136-154.

21. Lajeunesse D, Grimellec CL. 1984. Phosphate distribution and transport in mycoplasma. Can J Biochem Cell Biol 62:1041-1045.

22. Locke NA. 2015. Classification of polyphosphate-accumulating bacteria in benthic biofilms. Pennsylvania State University.

23. Mehlig L, Petzold M, Heder C, Günther S, Müller S, Eschenhagen M, Röske I, Röske K. 2013. Biodiversity of polyphosphate accumulating bacteria in eight WWTPs with different modes of operation. J Environ Eng 139:1089-1098.

24. Merzouki M, Delgenes J-P, Bernet N, Moletta R, Benlemlih M. 1999. Polyphosphate-accumulating and denitrifying bacteria isolated from anaerobic-anoxic and anaerobic-aerobic sequencing batch reactors. Curr Microbiol 38:9-17.

25. Mudd S, Yoshida A, Koike M. 1958. Polyphosphate as accumulator of phosphorus and energy. J Bacteriol 75:224.

26. Mullan A, Quinn J, McGrath J. 2002. Enhanced phosphate uptake and polyphosphate accumulation in *Burkholderia cepacia* grown under low-pH conditions. Microb Ecol 44:69-77.

27. Noegel A, Gotschlich EC. 1983. Isolation of a high molecular weight polyphosphate from *Neisseria gonorrhoeae*. J Exp Med 157:2049-2060.

28. O'Brien G, Harris J, Milnes A, Veeh H. 1981. Bacterial origin of East Australian continental margin phosphorites. Nature 294:442-444.

29. Peng L, Jiang Q, Pan J-Y, Deng C, Yu J-Y, Wu X-M, Huang S-H, Deng X-Y. 2016. Involvement of polyphosphate kinase in virulence and stress tolerance of uropathogenic *Proteus mirabilis*. Med Microbiol Immunol 205:97-109.

30. Qian Y, Borowski WJ, Calhoon WD. 2011. Intracellular granule formation in response to oxidative stress in *Bifidobacterium*. Int J Food Microbiol 145:320-325.

31. Rashid MH, Kornberg A. 2000. Inorganic polyphosphate is needed for swimming, swarming, and twitching motilities of *Pseudomonas aeruginosa*. Proc Natl Acad Sci U S A 97:4885-4890.

32. Reitz T, Merroun M, Selenska-Pobell S. 2007. Spectroscopic and microscopic studies on the interactions of *Paenibacillus sp*. JG-TB8 with U (VI). Institute of Radiochemistry: 41.

33. Seufferheld M, Vieira MC, Ruiz FA, Rodrigues CO, Moreno SN, Docampo R. 2003. Identification of organelles in bacteria similar to acidocalcisomes of unicellular eukaryotes. J Biol Chem 278:29971-29978.

34. Shi T, Ge Y, Zhao N, Hu X, Yuan Z. 2015. Polyphosphate kinase of *Lysinibacillus sphaericus* and its effects on accumulation of polyphosphate and bacterial growth. Microbiol Res 172:41-47.

35. Smart J, Robson A, Dilworth M. 1984. A continuous culture study of the phosphorus nutrition of *Rhizobium trifollii* WU95, *Rhizobium* NGR234 and *Bradyrhizobium* CB756. Arch Microbiol 140:276-280.

36. Streichan M, Schön G. 1991. Periplasmic and intracytoplasmic polyphosphate and easily washable phosphate in pure cultures of sewage bacteria. Water Res 25:9-13.

37. Takahashi N, Kalfas S, Yamada T. 1995. Phosphorylating enzymes involved in glucose fermentation of *Actinomyces naeslundii*. J Bacteriol 177:5806-5811.

38. Tanzer J, Krichevsky M. 1970. Polyphosphate formation by caries-conducive *Streptococcus* SL-1. Biochim Biophys Acta 215:368-376.

39. Tumlirsch T, Sznajder A, Jendrossek D. 2015. Formation of polyphosphate by polyphosphate kinases and its relationship to poly (3-hydroxybutyrate) accumulation in *Ralstonia eutropha* strain H16. Appl Environ Microb 81:8277-8293.
